# 

**Published:** 1894-12

**Authors:** 


### CONTENTS.

COMMUNICATIONS.

Dentistry Among the Speechless.... 573
Bridge-Work.... 576

PROCEEDINGS.

The American Dental Association.... 578
Vulcanizing Rubber.... 599

LEGISLATION.

Dental Law.... 601

SELECTIONS.

The Tongue in Disease.... 604
Purpura Hemorrhagica.... 605
Lawson Tait on Bacterial Pathology.... 606
Cocaine Anæsthesia Rendered Harmless by the Addition of Trinitrine. 606
Borax.... 606
Chloroform Narcosis.... 607
The Semi Centennial of the Discovery of Anæsthesia.... 607
Potassium Permanganate in Morphine Poisoning.... 608
The Absorption of Odors by Milk.... 609
Patent Medicines.... 609
The Probable Origin of the Red Blood-Corpuscles.... 609
A Method of Using Cocaine for Local Anæsthesia.... 611
Another Woman Honored.... 612
Women as Sanitary Inspectors.... 612
Rules Concerning What Patients Tell Experts.... 612

EDITORIAL.

A New Dental Journal.... 613
Mississippi Valley Dental Society.... 613
Bibliographical.... 614

